# Surface Science View of Perfluoroalkyl Acids (PFAAs) in the Environment

**DOI:** 10.1021/acsenvironau.3c00079

**Published:** 2024-04-30

**Authors:** Philip J. Brahana, Ruchi Patel, Bhuvnesh Bharti

**Affiliations:** Cain Department of Chemical Engineering, Louisiana State University, Baton Rouge, Louisiana 70803, United States

**Keywords:** PFAS, fluorinated surfactants, interfacial adsorption, pollutant transport, environmental fate, forever chemicals

## Abstract

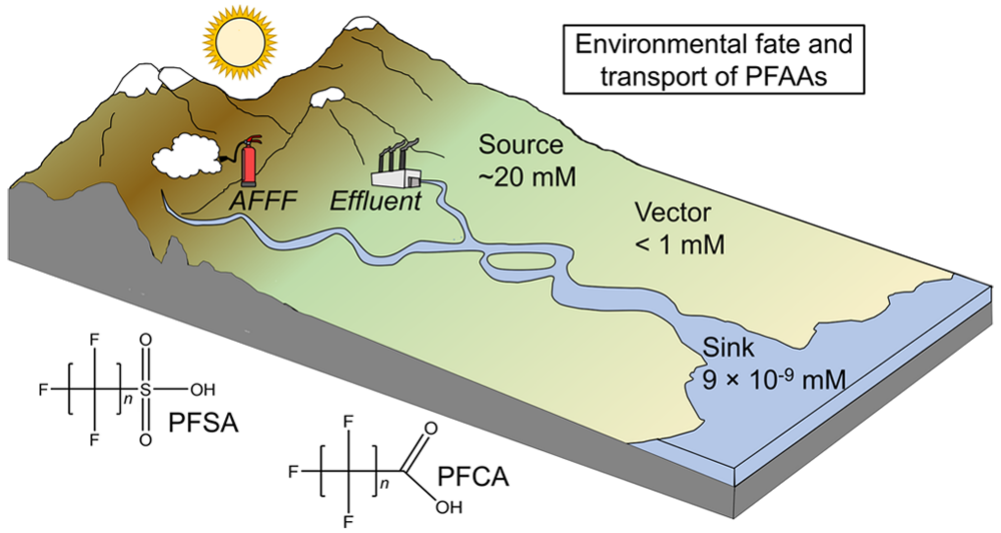

Per- and polyfluoroalkyl substances (PFAS) constitute a notorious category of anthropogenic contaminants, detected across various environmental domains. Among these PFAS, perfluoroalkyl acids (PFAAs) stand out as a focal point in discussions due to their historical industrial utilization and environmental prominence. Their extensive industrial adoption is a direct consequence of their remarkable stability and outstanding amphiphilic properties. However, these very traits that have made PFAAs industrially desirable also render them environmentally catastrophic, leading to adverse consequences for ecosystems. The amphiphilic nature of PFAAs has made them highly unique in the landscape of anthropogenic contaminants and, thereby, difficult to study. We believe that well-established principles from surface science can connect the amphiphilic nature of PFAAs to their accumulation and transport in the environment. Specifically, we discuss the role of interfacial science in describing the stability, interfacial uptake (air–liquid and solid–liquid), and wetting capability of PFAAs. Surface science principles can provide new insights into the environmental fate of PFAAs, as well as provide context on their deleterious effects on both the environment and human health.

## 1 Introduction

Per- and polyfluoroalkyl substances (PFAS) have been detected in environmental media, biological organisms and in the blood serum of nearly every human living in the industrialized world.^1^ The widespread presence of PFAS in the environment, coupled with their remarkable longevity, has earned them the epithet “forever chemicals”. The persistence of PFAS originates from their unmatched chemical stability and their remarkably slow degradation kinetics.^2^ PFAS encompasses a class of chemical compounds in which the hydrogen atoms present in hydrocarbon sections of molecules are substituted with fluorine atoms.^3^ One of the most notable and omnipresent forms of PFAS is perfluoroalkyl acids (PFAAs). PFAAs have been used for decades in firefighting foams, fluoropolymer production, and numerous manufacturing processes.^4^ However, widespread use and improper disposal of these chemicals have resulted in their deposition into the environment. PFAAs in the environment have a high degree of mobility, resulting in their accumulation in remote locations far from the source and integration within the ecological food chain.^5^ The former being their geographical distribution, and the latter referring to their ability to transfer through the food web and bioaccumulate in apex predators. While PFAAs are reported in many environments and biological organisms, their life cycle and potential impacts remain poorly understood. This lack of knowledge stems from the inherent complexity of the thermodynamic and transport characteristics of the PFAA molecules, which are highly dependent on the surrounding environmental conditions. To begin to address the existing knowledge gaps, this Perspective aims to link the research frontiers of surface science and environmental chemistry by discussing the mechanisms through which the fate of PFAAs in the environment is impacted by their unique amphiphilic and interfacial properties.

**Figure 1:**
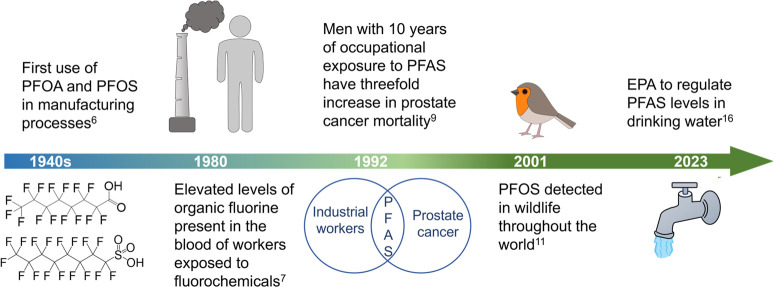
Timeline displaying some of the major events in the history of PFAS use. Timeline spans from the first use of PFOA and PFOS in the 1940s to the recent health advisories of PFOA and PFOS in drinking water, as determined by the EPA.

The development of PFAAs first began in the 1940s with perfluorooctanoic acid (PFOA) and perfluorooctanesulfonic acid (PFOS), compounds containing eight carbon atoms in their hydrophobic tail and an anionic carboxylic and sulfonic acid as the hydrophilic headgroup, respectively (Figure 1).^3,6^ At the time of their development, PFOA and PFOS were the most surface-active molecules that retained their amphiphilicity even under extreme conditions. These properties made PFAAs highly desirable in industry but ultimately led to an environmental catastrophe. Several decades later, concern arose for workers who were occupationally exposed to PFAAs and other forms of PFAS. Cohort studies revealed that individuals who were occupationally exposed to fluorochemicals had elevated levels of organic fluorine in their blood serum.^7^ The discovery generated keen interest within the scientific community to investigate the possible health risks associated with extended exposure to PFAAs. Eventually, epidemiological associations between exposure to PFAAs and prostate cancer mortality,^8^ hepatotoxicity,^9^ and lower infantile birthweight^10^ were illuminated, and their presence was reported in wildlife throughout the world.^11^ Increasing scientific evidence has highlighted the underlying link between PFAAs and their detrimental effects to the health of both wildlife and humans.^8,12−15^ As of 2023, the US Environmental Protection Agency (EPA) is set to regulate the levels of six different types of PFAS (including PFOS and PFOA) in the US drinking water reservoirs.^16^ While the efforts are underway to find benign alternatives to the PFAAs, the Center for Disease Control (CDC) has revealed that PFAS are found in nearly every US citizen, and their use continues worldwide.

**Figure 2:**
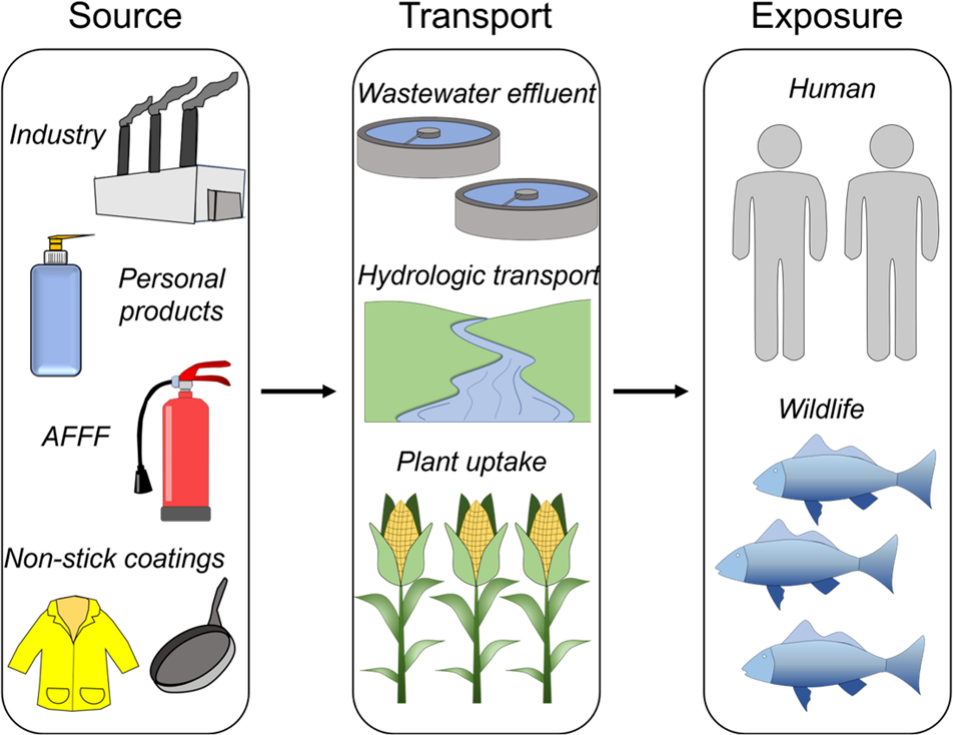
Environmental mobilities of PFAAs. Point sources of PFAAs in the environment can include manufacturing processes, personal products, firefighting foams, and nonstick coatings. After their environmental deposition, PFAAs can transport through pathways of wastewater effluent, rivers, and streams and even plant uptake, before eventually accumulating in biological organisms.

PFAAs exhibit great environmental mobility, traversing through different compartments before ultimately accumulating in biological organisms (Figure 2). At sites which have been treated with aqueous film forming foam (AFFF), PFOS has been reported as the predominate PFAA present.^17,18^ However, in other environmental matrices like surface runoff, air, snow, rain, etc., PFOA is often reported as the most prevalent PFAA.^19^ PFAAs have been reported in wastewater effluent^20^ and rivers,^21^ and have been shown to accumulate in plants.^22^ Through their extensive environmental mobility and accumulation pathways, humans and wildlife can ultimately be exposed to these persistent organic pollutants (POPs). Historically, the presence and transport of POPs in the environment has been well-reported.^23^ A vast literature exists which describes the adverse environmental effects and atmospheric transport of common POPs, such as polychlorinated biphenyls (PCBs),^24,25^ organochlorine pesticides^26^ and other organic pollutants. PFAAs possess distinct characteristics among POPs due to their elevated interfacial activity and their ability to withstand degradation. Therefore, assessing the issue of PFAA pollution through the same framework as for other common anthropogenic pollutants is insufficient. Similar to other environmental issues such as microplastics^27,28^ and oil spills,^29,30^ colloid and surface science can play a central role in bridging knowledge gaps on the respective issues.

In this Perspective, we aim to highlight the underlying links in the research fields of environmental science and interfacial science and present a “beginner’s guide” to fundamental surface science in the context of PFAAs in the environment. The concepts presented in this Perspective can enrich our understanding of PFAAs behavior in the environment. In order to gain a deeper comprehension and anticipate the life cycle of PFAAs, the Perspective will utilize the principles of surface science, including: (1) The physiochemical properties of these compounds, in relation to their functionality; (2) their capacity for interfacial binding at air–liquid and solid–liquid interfaces; and (3) their capability to modify the wetting characteristics of surfaces. The application of these concepts will provide a holistic framework to fill knowledge gaps in our understanding of the thermodynamic and transport properties of the PFAAs both in the environment and in the human body.

## 2 Why Are “Forever Chemicals” Forever?

**Figure 3:**
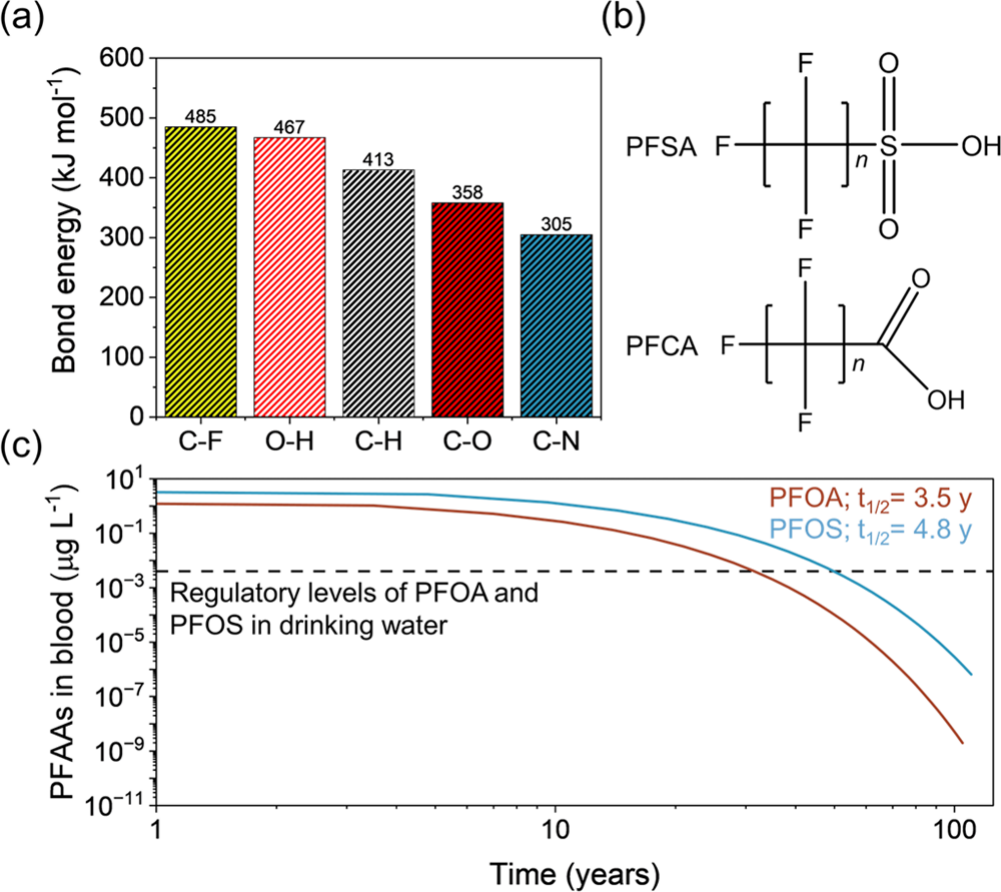
PFAAs as forever chemicals. (a) Bar graph comparing different bond energies obtained from literature,^35^ demonstrating the stability of the C–F bond relative to other common chemical bonds. (b) Chemical structures of perfluorooctanesulfonic acid (PFSA) and perfluorocarboxylic acid (PFCA). (c) Elimination kinetics of PFOA (red curve) and PFOS (blue curve) in human blood serum, assuming typical excretion pathways and no new exposure. The dotted line represents the proposed regulatory levels of the two PFAAs in drinking water defined by the EPA in 2023.

The release of surfactant waste into the environment is not a new occurrence, and the technologies for eliminating residual surfactant waste from effluent streams has been widely discussed.^31−33^ One of the historical approaches to surfactant effluent remediation has been to allow nature to take its course through biodegradation. While natural degradation may be an appropriate waste management approach for hydrocarbon-based surfactants, it is not suitable for PFAAs due to their unmatched stability. The stability of PFAAs originates from the inherent nature of the carbon–fluorine bond present in the molecules. The C–F bond is often referred to as the “*most stable bond in organic chemistry*”, attributed to the high electronegativity of fluorine.^34^ The electronegativity of fluorine yields a large dipole moment of the C–F bond and a high bond dissociation energy of 485 kJ mol^–1^, which is considerably higher than other common chemical bonds (Figure 3a).^35^ These C–F bonds make up the hydrophobic tail of the PFAAs (Figure 3b) and are formed through the electrochemical fluorination of their hydrocarbon counterparts. Electrochemical fluorination is a process in which an organic material undergoes electrolysis in the presence anhydrous HF, replacing all hydrogen atoms with fluorine atoms. For the synthesis of PFAAs, hydrogen atoms in the chain of a hydrocarbon surfactant are substituted with fluorine atoms.^36^ The stability of the C–F bond enables the PFAA to remain functional in extreme temperatures and chemical conditions that would quickly deteriorate traditional surfactants.^37^

The high stability of the C–F bond has enabled PFAAs to find application in many industrial processes and is simultaneously the root cause of the environmental catastrophe at hand.^3,4,38^ The slow elimination kinetics of PFAAs from the human body is demonstrated in Figure 3c. The mean half-life (*t*_1/2_) of PFOA and PFOS in the blood serum of occupationally exposed workers was experimentally determined to be 3.5 and 4.8 years, respectively.^39^ This half-life is the time period in which the concentration of the PFAAs drops to half of its original concentration as molecules are excreted from the serum. As of the 2017–2018 National Health and Nutrition Examination Survey (NHANES), the average concentration of PFAAs in blood serum of the public is ∼1.4 and 4.3 μg L^–1^ for PFOA and PFOS, respectively.^40^ A rough estimation of the elimination of the PFAAs currently present in the serum of American citizens can be done by assuming first order kinetics. The calculations show that it would take several decades for an individual who possesses above-mentioned concentrations of PFOA and PFOS to expel them from their blood serum (Figure 3c). The exact mechanisms by which PFAAs are excreted from the blood serum are still debated, but is likely attributed to a combination of urinary and biliary excretion, gut resorption or variation in isomer absorption in the gastrointestinal tract.^39,41−43^ It is to be recognized that the nondegrading nature of PFOA and PFOS remains a fundamental issue, as clearly demonstrated by these simple estimates of elimination kinetics. In fact, the extremely long retention time of the PFAA molecules is the primary cause of its human health concerns, as the prolonged presence of these molecules in human body could increase the possibility of being afflicted with severe diseases, including cancer.^12,44,45^

The surface activity and amphiphilicity of PFAAs are highly dependent on the length of its fluoroalkyl tail and dissociated state of the headgroup.^46,47^ The behavior of a PFAA molecule can vary considerably based on its hydrophilic headgroup, which could in turn influence micellization, aggregation and solubility.^33^ One of the most notable classes of PFAAs is perfluorocarboxylic acids (PFCAs), which feature a hydrophilic headgroup that consists of a carboxylic acid (Figure 3b). At weakly acidic pH, the carboxylic acid headgroup of PFCAs begins to dissociate, leading to the formation of a negative charge and thus impacting the amphiphilicity of the molecules (discussed in section 3.1). Additionally, the headgroups of PFAAs have been reported to play a critical role in their chemical reduction in the presence of hydrated electrons.^48^ One study has shown the differences in the hydrated electron-induced reduction kinetic pathways of PFAAs with a carboxylate headgroup (PFCAs) and a sulfonic headgroup (PFSAs).^49^ In the case of PFCAs, the α-position carbon atom adjacent to the carboxyl group is the primary target for the binding of hydrated electrons.^50^ This can be attributed to the inductive effect of the anionic headgroup. Whereas *in vitro* studies on the degradation of PFSAs have found that the degradation reaction pathways are distinct from PFCAs, and can include desulfonation, H/F exchange and chain shortening via C–C cleavage.^50^ In desulfonation, reduction of the fluoroalkyl chain does not occur until the sulfonic headgroup is transformed into a carboxylic acid. The cleavage of the C–S bond readily occurs due to the lower bond energy of the C–S bond (272 kJ mol^–1^) relative to the C–C bond (346 kJ mol^–1^) in the fluoroalkyl chain.^50^ The desulfonation process occurs when the attachment of hydrated electrons breaks the C–S bond between the headgroup and the fluoroalkyl chain. After scission of the C–S bond, the carbon atom at the headgroup becomes oxidized into carboxylic acid, forming a PFCA. From this point, H/F exchange or chain scission occurs and the PFAA degradation continues.^50^ In a natural setting, the desulfonation of PFSAs is plausible under the action of microbial activity.^51,52^ However, the microbial desulfonation of PFSAs is limited to environments that are low in sulfur, as microorganisms in sulfur-rich environments tend to favor other compounds, such as sulfate and sulfur-containing minerals.^53^

Under the action of microbial activity, the reductive defluorination of perfluorinated compounds can occur in nature.^51,52^ However, the differences in the efficiency of the model bacteria to reduce PFAAs with dissimilar headgroups are yet to be identified. It should be noted that successful cases of microbial degradation of PFOA and PFOS are seldom found in current literature. Studies which have identified conditions conducive to the microbial degradation of other PFAS found in AFFFs report degradation products comprising shorter-chained fluorinated surfactants (typically C < 6).^54^ Additional studies have reported the natural microbial degradation of these shorter-chained fluorinated compounds, highlighting the potential use of microorganisms in the remediation of PFAS. The isolation of the specific enzyme responsible for defluorination of PFAAs by microorganisms is crucial for bioremediation to become a viable method.

## 3 Interfacial Uptake and Adsorption of PFAAs

The extensive use of PFAAs as industrial surfactants is due to their superior interfacial activity over their hydrocarbon counterparts.^4^ The adsorption of PFAAs on to interfaces is critical in governing their environmental accumulation, transport, and toxicity. Here, we will discuss mechanisms of PFAA adsorption at (1) air–liquid and (2) solid–liquid interfaces. The aim of this section is to link fundamental concepts of surfactant science to the potential environmental and adverse health impacts of the PFAAs.

### 3.1 Adsorption at the Air–Liquid Interface

**Figure 4:**
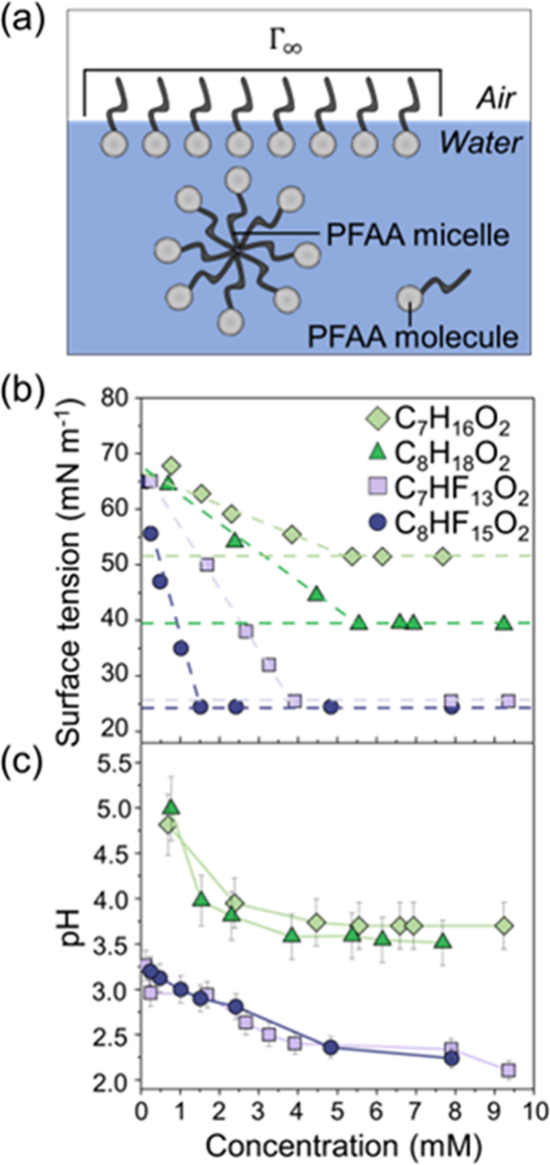
PFAA adsorption at the air–liquid interface. (a) Schematic representations of surfactant molecules in the aqueous solution, Γ_∞_ is the maximum surface excess. (b) Experimental measurements of the surface tension of aqueous solutions containing PFCAs (PFHpA and PFOA) and fatty acids (heptanoic and octanoic acid). The lines are linear fits to the data for surfactant concentrations above and below the cmc of the respective surfactant. (c) pH values of the solutions containing the model PFCAs and fatty acids as a function of their aqueous concentrations. All symbols represent experimental measurements; error bars in (c) signify the uncertainties in the pH measurements.

To fully understand the environmental fate of PFAAs, it is necessary to consider their behavior at the boundary between the two immiscible phases. The interface between two phases has high energy, which governs unique chemical and physical phenomena. One such aspect is the interfacial tension, which is the free energy change in expanding the interface by a unit area.^55^ Surfactants are known to reduce the interfacial free energy of two immiscible phases by adsorbing at the interface (Figure 4a). However, the efficiency of this process varies among surfactants. PFAAs, in particular, exhibit a significantly higher affinity for the air–liquid interface compared with their hydrocarbon counterparts (Figure 4b). The lowering of the surface tension upon using fluorinated surfactants is due to the increase in their interfacial adsorption. The surface excess concentration of the PFAAs accumulated at the air–water interface (Γ), is given by Gibbs adsorption equation as^46^
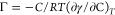
1where *C* is the surfactant concentration, *R* is the gas constant, γ is the surface tension (mN m^–1^), and *T* is the temperature. This equation relates the surface concentration of surfactants to the surface tension and can be used to quantify the extent of PFAA adsorption at the air–water interface.^56,57^ The maximum surface excess (Γ_∞_) can then be calculated from the slope of the surface tension vs surfactant concentration curve below the critical micelle concentration, i.e., cmc (Figure 4b). Furthermore, by applying the Gibbs adsorption equation to the experimental data presented in Figure 4b, one can estimate other properties of the surfactants, including maximum surface excess (Γ_∞_) and air–water partitioning coefficient.^46^

To exemplify the critical role of the fluorinated tail in interfacial adsorption, we measure the surface tension of two model PFCAs, perfluoroheptanoic acid (PFHpA) and PFOA and two model fatty acids, heptanoic acid and octanoic acid. In our experiments, elevated surface activity is observed for the model PFCAs in comparison to their fatty acid counterparts with an identical number of carbon atoms in the tail. Furthermore, we report a decrease in the cmc for the model PFCAs relative to those of the fatty acid molecules, indicative of heightened chain–chain attraction. Finally, a discernible reliance on the hydrophobic tail length is identified, influencing both the cmc and surface activity of the respective molecules. It should be noted that we observe a decrease in the pH as a function of concentration for both the fatty acids and PFCAs (Figure 4c). The extent of interfacial adsorption of PFCAs would thus depend on the changes in the pH of the solution driven by ionization of the PFCA headgroups, as in the case of fatty acids.^58^ The results from this experiment describe the outstanding surface properties of PFCAs relative to their fatty acid counterparts while also suggesting careful examination of the pH of the media as a critical parameter when predicting the fate and transport of PFCAs in the environment.

Similar to the pH of the media, the p*K*_a_ of a molecule is an important parameter to consider when conducting experimental research. The p*K*_a_ is a fundamental parameter that represents the pH at which the concentration of the molecules in the dissociated (ionized) and undissociated (nonionized) states are equal. In other words, it describes the propensity of a molecule to donate or accept protons, ultimately determining its behavior such as interfacial activity for PFCAs. The p*K*_a_ value of a molecule is strongly dependent on the local chemical environment. In traditional surfactant science, it is critical to differentiate the p*K*_a_ of surfactant molecules at an interface (air–liquid, solid–liquid) from the molecules present in bulk of the solution, as it will govern the physiochemical properties of the molecules.^59,60^ This phenomena has been studied in literature regarding surface-active fatty acids,^58,61^ and has only recently been demonstrated for PFCAs.^62^ The electron-withdrawing nature of perfluoroalkyl groups in PFCAs, as opposed to the electron-donating characteristics of alkyl groups in fatty acids, points to the potential for unique alterations in their interfacial behavior. Through a series of pH titrations, we recently discovered that the p*K*_a_ values of different PFCAs in an aqueous solution vary depending on whether the PFCA molecules are adsorbed at the solution interface or present in the bulk.^62^ This examination of the surface-p*K*_a_ of PFCAs assists in addressing the discrepancies in the reported p*K*_a_ values of PFCAs.^48,63−65^ The determination of the surface-p*K*_a_ of PFAAs at the air–water interface is thus a critical parameter that dictates the pH at which protonation and deprotonation transpire. This, in turn, would influence the interfacial activity of PFAAs, leaving the potential for far-reaching environmental consequences. Specifically, existing literature describes the critical role of surface-p*K*_a_ of model fatty acids in impacting environmental phenomena such as foamability,^66^ evaporation rate,^67^ droplet lifetime^68,69^ and the nucleation activity of sea spray aerosols (SSAs).^70^ However, more comprehensive studies that focus on the interplay between surface tension, pH, and concentrations of PFAAs hold the potential to improve environmental assessments of PFAAs, yielding consistent experimental conclusions.

Accumulation of PFAAs at the air–liquid interface can have significant implications for (1) interfacial tension, (2) interactions with other molecules and compounds, and (3) environmental transport processes. The experiments presented above are not meant to mimic environmental conditions but rather to shed light on the dynamic surfactant properties of PFAAs in different aqueous conditions. Additionally, we demonstrate the role of PFAAs in reducing the interfacial free energy, which can affect phenomena such as droplet spreading, wetting, and emulsification. In the environment, PFAAs can also interact with organic and inorganic matter present at interfaces,^71,72^ including dissolved ions^73^ and other contaminants.^74^ Understanding the competition or cooperativity in the adsorption process due to the interactions of PFAAs with other substances at the air–liquid interface is crucial for assessing their environmental behavior. Furthermore, the adsorption of PFAAs at air–liquid interfaces is interconnected with their volatilization potential i.e. their ability to volatize from the liquid phase into the surrounding atmosphere as a major component of aqueous aerosols, which will be further discussed in section 4.1.^75,76^ Similarly, PFAA concentration and their adsorption onto solid–liquid interfaces, such as in soil, can also influence their environmental transport.^77^

### 3.2 Adsorption at the Solid–Liquid Interface

The amphiphilic nature of the PFAAs allows for their adsorption at solid–liquid interfaces.^78^ Anionic PFAAs are capable of adsorbing onto an oppositely charged substrate via electrostatic attraction between the surface and the headgroup. Additionally, the hydrophobic tail of PFAAs enables their adsorption onto nonpolar substrates via hydrophobic attraction between the substrate and the fluorinated tail. This section will primarily focus on the latter, due to its high relevance in the adsorption of anionic PFAAs on environmentally relevant surfaces such as soil,^79^ as well as its role in remediation technologies.^80^

Hydrophobic interactions refer to the attraction between hydrophobic domains and molecules in an aqueous solution. These interactions are driven by the gain in entropy of water molecules released upon the association of hydrophobic regions/molecules in the aqueous solvent.^81^ In the context of PFAAs, hydrophobic interactions can facilitate their adsorption onto hydrophobic solid–liquid interfaces.^78^ More specifically, the hydrophobic tail of the surfactant may attach onto the surface, while the hydrophilic headgroup points toward the solvent.^82^

**Figure 5:**
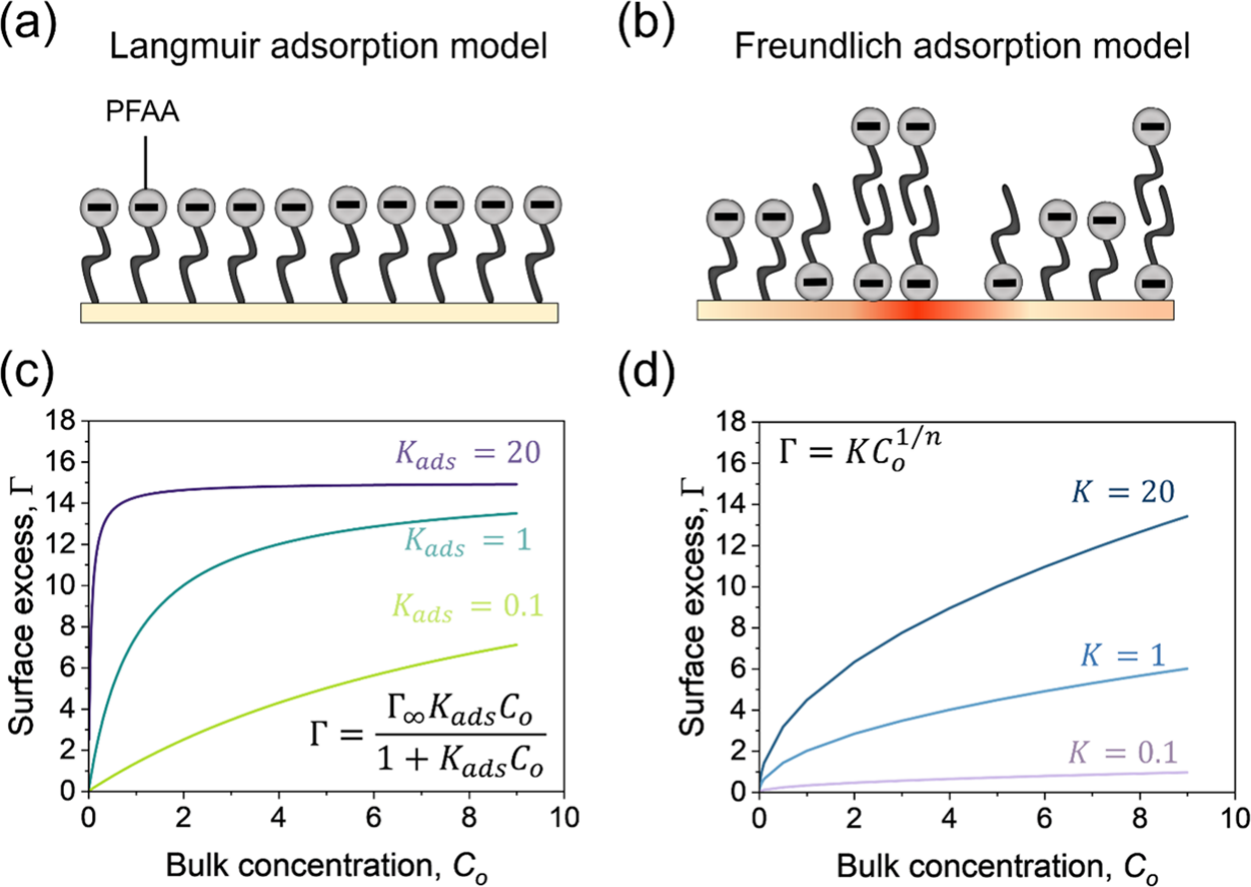
PFAA adsorption at the solid–liquid interface. Schematic representation of adsorption according to the Langmuir (a) and Freundlich (b) models. Representative adsorption isotherms were modeled using the Langmuir (c) and Freundlich (d) equations with increasing adsorption constants.

The adsorption of PFAAs at a solid–liquid interface is influenced by both the chemical structure of the surfactant (i.e., headgroup composition and fluoroalkyl chain length) and the surface characteristics of the adsorbent (i.e., charge, surface area, and chemistry). These parameters govern the adsorption behavior of PFAAs onto a solid substrate, which can be quantified by using adsorption isotherms. Two frequently used models to describe the adsorption behavior of PFAAs onto a solid substrate are the Langmuir and Freundlich models. The Langmuir model assumes monolayer adsorption of molecules onto a surface with a finite number of sites with identical binding energy, ε (Figure 5a). It gives the surface excess at the solid–liquid interface as^83^
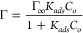
2where Γ_∞_ is the maximum surface excess at the solid–liquid interface, *K*_*ads*_ is the adsorption constant, and *C*_*o*_ is the bulk concentration of PFAA in solution at equilibrium.^84,85^ Γ_∞_ can be influenced by both the chemical structure of the adsorbate and the surface properties of the adsorbent. Correspondingly, the physicochemical relation between the adsorbing molecules and the surface governs the adsorption free energy and thus *K*_*ads*_ (discussed below). In fact, we recently demonstrated that the ability of PFCAs to adsorb onto microplastics was dependent on the fluoroalkyl chain length of the PFCA, as well as the surface charge and hydrophobicity of the microplastic substrate.^86^ The value for *K*_*ads*_ changes based on the binding affinity of the PFCA molecules for an adsorbent (Figure 5c). When the binding affinity is high, it results in a larger *K*_*ads*_ value, indicating robust interaction and greater adsorption. On the other hand, a lower binding affinity leads to a reduced *K*_*ads*_ value, signifying weaker binding and limited adsorption capacity. This relationship points to the influence of molecular interactions in surface adsorption processes with specific implications for the hydrophobic interaction between PFAAs and a solid substrate.

Contrasting the Langmuir model for adsorption, the Freundlich model is applicable for multilayer adsorption on a surface with a heterogeneous distribution of adsorption energies (ε_*i*_) as shown in Figure 5b. Mathematically, the expression for Freundlich adsorption isotherm is given as^33^
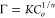
3where *K* is the Freundlich constant and *n* is the measure of the nonlinearity in the adsorption isotherm and dependent on the molecule–substrate interactions. The Freundlich isotherm can be used to describe the ability of the surface to uptake an adsorbate, based on the physicochemical properties of the surface. The Freundlich isotherm is a purely empirical model where the *K* is effectively a partition coefficient representing the adsorption capacity of the adsorbent, rather than the energy of the adsorption. This is the reason why the Freundlich model is applicable generically to the adsorption at interfaces but does not incorporate underlying thermodynamics of the process, therefore limiting its ability to describe the energetics of a system. The value of *K*, as determined through the Freundlich model, is sensitive to the binding affinity between the PFAA molecule and the adsorbent (Figure 5d). A high binding affinity results in a larger *K* value, indicating that the adsorbent’s capacity to capture and retain the PFAA is relatively high. In contrast, a lower binding affinity corresponds to a smaller *K* value, representing diminished adsorption capacity and a weaker interaction between the PFAA molecule and the adsorbent. While the Freundlich model sheds light on the capacity for adsorption of the adsorbent; it is essential to note that the Langmuir constant *K*_*ads*_, derived from the Langmuir model, offers thermodynamic insights. Under the previously described assumptions, *K*_*ads*_ is directly related to the Gibbs free energy of adsorption Δ*G*_*ads*_ and is often used to describe the spontaneity of the process.

The energetics of the adsorption process can be described by the Gibbs free energy of the adsorption. The Gibbs free energy can quantify the amount of energy released or gained when an adsorbate (e.g., PFAA) interacts with a solid substrate. The free energy of adsorption can be estimated with the experimentally obtained adsorption constant *K*_*ads*_ derived from the Langmuir model as^85,87,88^
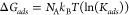
4where *k*_B_ is the Boltzmann constant and *N*_A_ is Avogadro’s number. Using these models, we can (1) study the interactions of PFAAs with environmentally relevant surfaces; (2) gain insights into the driving forces of PFAA adsorption/uptake, and (3) predict the adsorption behavior of PFAAs of various chain lengths (and correspondingly different amphiphilic properties) onto environmental media.

Quantifying the thermodynamic relationship of the adsorption of PFAAs onto environmental media can provide insight into their fate and transport. The Gibbs free energy not only describes the spontaneity of the adsorption process but also serves as a measure elucidating the partitioning behavior of PFAAs in various environmental compartments, including water, air, and soil. When PFAAs are introduced into the environment, they exhibit a tendency to distribute among these different environmental compartments, potentially resulting in their long-range transport. The partitioning behavior of PFAAs can often be correlated to their chemical structure. Specifically, PFAAs with longer fluoroalkyl chains tend to be more hydrophobic and, therefore, have a higher affinity for organic matter. However, short chain PFAAs are more water-soluble and are likely to remain in the aqueous phase.

## 4 Impacts of Interfacial Adsorption

### 4.1 Adsorption at the Air–Liquid Interface: Aerosolization and Transport

**Figure 6:**
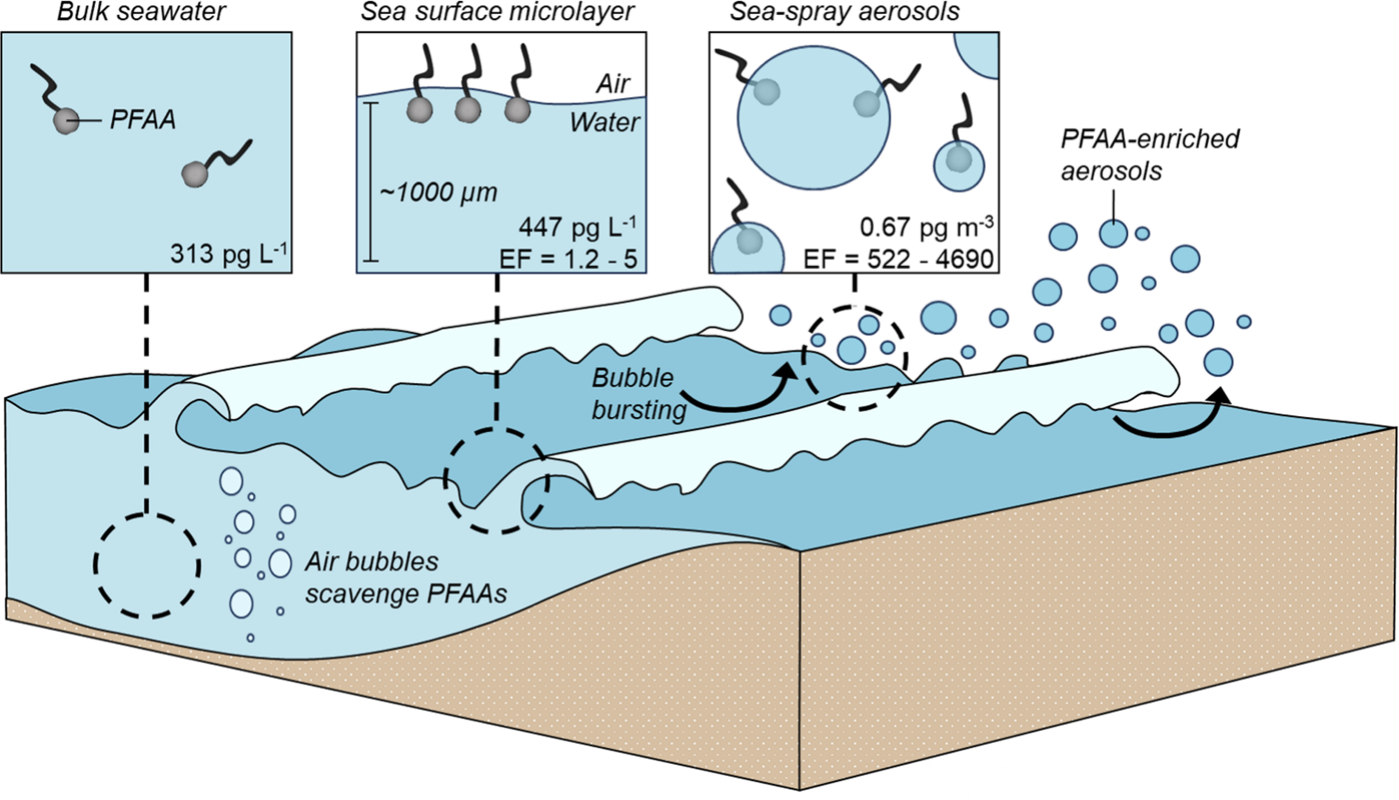
Aerosolization of PFAAs. Schematic which describes (1) the formation of SSAs in the ocean and (2) the concentrations of PFAAs reported in different oceanic compartments according to Casas et al.^76^ Concentrations are reported as picograms per liter of seawater or picograms per cubic meter of seawater (sea-spray aerosols). EF is the enrichment factor (see the text for details).

The adsorption of PFAAs at the air–liquid interface can facilitate their aerosolization and long-range environmental transport.^76,89,90^ The adsorbed state of PFAA molecules at the air–liquid interface has impacts on their migration from the liquid-phase to the gas phase (i.e., volatilization) and their transport to remote environments.^19^ In the context of the open ocean, aerosolization of PFAAs occurs through a multistep process (Figure 6). In the ocean, air bubbles are generated through various mechanisms, including turbulent water and breaking waves.^91,92^ When PFAA molecules are present in oceanic waters, air bubbles will “scavenge” the PFAAs as they migrate toward the ocean-atmosphere interface.^76,89^ Once at the ocean-atmosphere interface, there is a boundary referred to as the sea-surface microlayer (SML). The SML is reported to be approximately 1000 μm in thickness and contains a higher concentration of PFAAs relative to the bulk ocean water (Figure 6).^76,93^ This finding can be explained by both the affinity of PFAAs for the air–water interface and the transport of the PFAA molecules by oceanic air bubbles. However, the SML is not likely to be a terminal sink for the oceanic PFAA molecule. When waves and turbulence are active, small droplets containing PFAAs are propelled into the air. In a field study, Casas et al. reported the concentration of PFAAs in sea spray aerosols to be 0.63 pg m^–3^, with an enrichment factor (EF) of ranging between 522 and 4690 (Figure 6).^76^ In this instance, the EF is a unitless number that is calculated in order to compare the concentration of PFAAs in different oceanic compartments (SML and SSAs) relative to a background concentration (bulk seawater). As these PFAA-enriched water droplets rise into the atmosphere, they evaporate, leaving behind the PFAA molecules. These PFAA molecules can then interact with other airborne particulate matter, such as dust, to form PFAA-enriched aerosols.^76,94,95^ What happens to these PFAA-enriched aerosols after their formation is still uncertain. However, they could potentially contribute to cloud formation, influence weather patterns, or be transported over long distances to remote areas.

SSAs have been shown to influence ice nucleation and cloud formation over marine environments.^96^ This phenomenon has been described by classical nucleation theory (CNT). Nucleation can either be classified as homogeneous or heterogeneous, the former occurring in a pure environment while the latter occurs in the presence of foreign particles, surfaces or impurities.^97^ Heterogenous nucleation is highly relevant in a real-world scenario when compared to homogeneous nucleation. Heterogenous nucleation is a complex process influenced by factors such as supersaturation, temperature, and the chemical properties of the molecules involved. Using CNT, a fundamental framework for estimating the change in Gibbs free energy associated with heterogeneous nucleus formation at absolute temperature, given as
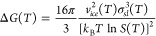
5where *v*_*ice*_(*T*) is the volume of a water molecule in ice (cm^3^), σ_*sl*_(*T*) is the interfacial tension between water and the ice embryo, and *S*(*T*) is the ice saturation ratio.^98^ Note that “ice embryo” refers to the small ice crystals that initially form when water vapor or liquid water transitions to the solid phase. A key aspect of the CNT and the corresponding Gibbs free energy is the interfacial tension between water and the ice embryo. In the presence of highly surface-active PFAAs, we can anticipate a decrease in the interfacial tension of the system. Although further theoretical and experimental research is needed to confirm this hypothesis, we expect that PFAA-enriched aerosols change the Gibbs free energy of the nucleation process. The alteration of Gibbs free energy, according to the CNT, could induce nontrivial impacts on atmospheric ice nucleation and cloud formation over marine environments.

### 4.2 Adsorption at Solid–Liquid Interface: Wettability Alteration

**Figure 7:**
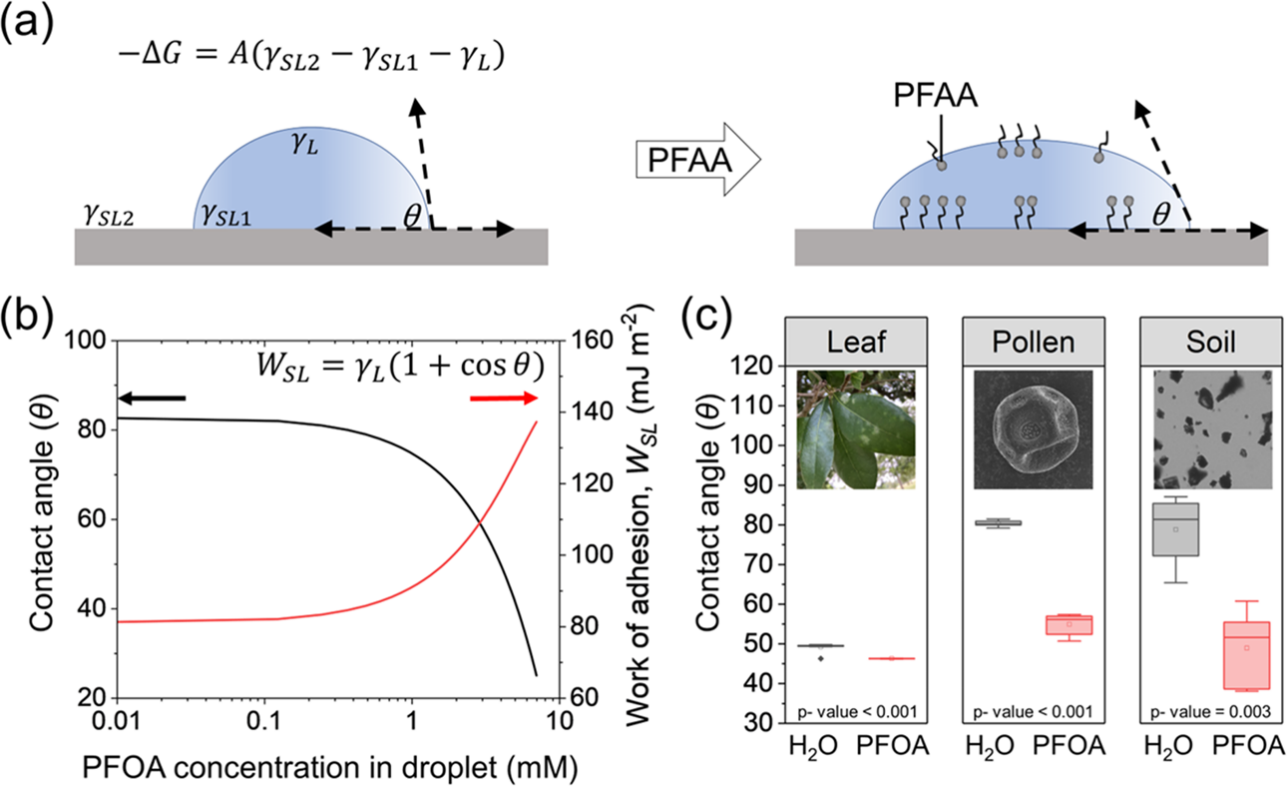
Influence of PFAAs on adhesion and wetting phenomena. (a) Schematic representation of how PFAAs can alter the wetting properties of a solid substrate, along with the equation representing the free energy change in the wetting process. (b) Representative plot describing the relationship between contact angle (left *y*-axis), work of adhesion (right *y*-axis), and PFAA concentration within the wetting aqueous droplet. (c) Experimental measurements of the contact angle of DI water (black) and DI water containing PFOA (red) on various environmental media. The box plot displays the upper and lower quartiles of the data with the box edges, and the median is shown as a horizontal line inside the box. The mean value is represented by a point within each box, and the whiskers extend to show the minimum and maximum measured values for each experiment. The *p*-values in the figure were calculated from an unpaired *t* test (*t*_leaf_(10) = −8.9, *t*_pollen_(10) = −26, and *t*_soil_(10) = −4.5); data sets passed the Shapiro-Wilks test for normality.

PFAAs have the potential to modify the wetting characteristics of a solid substrate by adsorbing at the solid–liquid interface. The thermodynamic implications of surface wetting properties extend beyond surface characterization and include fundamental concepts such as surface energy,^99^ work of adhesion, and droplet spreading.^55^ The process of wetting is when a fluid (water) and a solid are in contact, and this fluid subsequently spreads to displace a second fluid (air). As wetting takes place, the interfacial area between the solid and second fluid (air) decreases, while there is a corresponding increase in the interfacial contact area between the solid and the first fluid (water). The total energy change in the system is given by −Δ*G* = *A*(γ_*SF*2_ – γ_*SF*1_ – γ_*F*_) (Figure 7a), where γ_*SF*1_ and γ_*SF*2_ refer to the solid–liquid interfacial energies for the two fluids, γ_*F*_ is the interfacial tension between fluids one and two, and *A* is the total surface area of the substrate.^33^

The way PFAA molecules adsorb at the solid–liquid interface plays a crucial role in influencing the wetting properties of the underlying solid-substrate. Specifically, on a hydrophobic surface, the PFAA molecule attaches itself via its hydrophobic tail, thus exposing its hydrophilic headgroup toward the solvent.^33,86^ This orientation holds significance, as it can impact the wettability of the surface, leading to a reduction in its contact angle and a consequent increase in its hydrophilic character (Figure 7a). PFAA induced alterations to the wettability of environmental media can impact their transport properties and interactions with other components in the ecosystem, ultimately affecting natural environmental cycles.

Imagine a forest that has experienced wildfire and has been treated with AFFF containing PFAAs. Legacy PFAAs from AFFFs could affect ecological cycles within a forest by altering the wettability and surface properties of forest components. PFAAs present in the AFFF can alter the water wettability of environmental surfaces, which is inversely proportional to the work of adhesion *W*_*SL*_ between liquid and a solid substrate (Figure 7b). Traditionally, work of adhesion has been used as a measure of the interaction between a solid substrate and a surfactant solution, providing insights into the adhesion strength and the efficiency of surface modification techniques.^75,100,101^ In a classic example, Pashley and Israelachvili calculated the work of adhesion between a (1-Hexadecyl) trimethylammonium bromide (CTAB) solution on a mica surface, demonstrating that the concentration of the CTAB surfactant had a clear effect on the magnitude of *W*_*SL*_ required to separate the solid–liquid interface.^102^*W*_*SL*_ can be calculated as a function of the contact angle (θ) through the Young–Dupré equation, which is given as
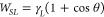
6where γ_*L*_ is the surface tension value for liquid phase.^102^ The magnitude of the work of adhesion between two contacting phases is directly proportional to the strength of the intermolecular interactions at their interface and, thereby, signifies the degree of attraction between them. In Figure 7b, we propose a hypothetical scenario where PFOA adsorbs to a surface via its hydrophobic tail, leaving its hydrophilic headgroup facing outward toward the solvent (as described in section 3.2). In this case, the contact angle will decrease as PFOA molecules populate the surface, and there will be a corresponding increase in *W*_*SL*_. To demonstrate this, we use a goniometer to experimentally obtain contact angle values for various environmental media with both pure water and water containing PFOA (Figure 7c). Here, we provide preliminary evidence that PFAA concentrations which are typically reported in the literature (∼25 ng L^–1^) can significantly alter the wettability and work of adhesion of various environmental media, including topsoil, tree pollen (*Liquidambar styraciflua)*, and tree leaves (*Quercus virginiana*). In our experimental findings, we observe that the influence of PFOA on pollen was particularly significant, with an observed reduction of ∼25% in contact angle upon exposure to PFOA, and a corresponding increase in the work of adhesion. These findings are attributed to the inherent hydrophobicity of the pollen samples, which engenders a more robust interaction between the hydrophobic tail of PFOA and the surface of the pollen.

From an environmental science perspective, understanding wettability and the consequent changes in the work of adhesion are important for several reasons. First, it can illuminate the mechanism by which contaminants interact with environmental media, such as soil particles or plant leaves. Contaminants that increase the work of adhesion between a solid surface and water, such as PFAAs, are more likely to bind and remain in place, potentially leading to environmental and health concerns. Second, the alteration in the work of adhesion resulting from adsorption-induced changes to wettability may have far-reaching consequences for the transport properties of environmental particles, including pollen. The transport of pollen, via adhesion, is a critical process that has direct implications for the reproductive success and survival of plant populations within a forest ecosystem.^103^ Thus, any alteration to the transport properties of pollen, including those induced by changes in wettability, could have cascading effects on the ecology of the entire forest ecosystem. Finally, an increase in the wettability of environmental media, such as plants, can modify the rates of condensation and droplet nucleation. In other words, water droplets will tend to form more readily on surfaces that display a greater hydrophilic character.^104^ Consequently, in certain plants, where the wettability of their leaf surface plays a crucial role in their water absorption process, it can be expected that any changes to the leaf wettability could affect the well-being of these plants. The wettability and work of adhesion of environmental media can have further implications for the environmental fate of PFAAs, which is an important consideration for their transport into other ecosystems.

## 5 Rethinking the Existence of PFAAs in the Environment

**Figure 8:**
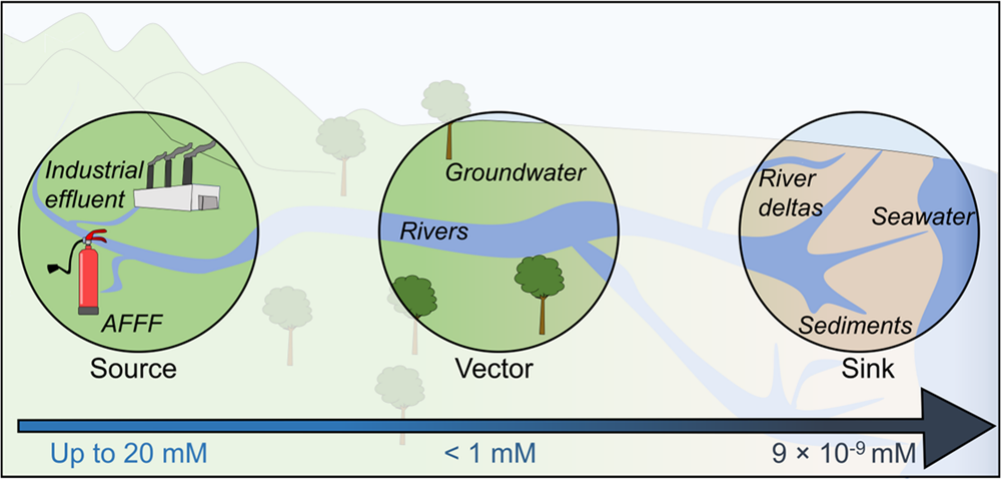
PFAS in the environment. Schematic representation of examples of different environmental sinks for PFAS contamination as a function of reported environmental concentrations. Sources include AFFF and industrial effluence, vectors of transport including surface and groundwater,^106,109^ and temporary sinks such as seawater.^110^

PFAAs have been detected in the environment at a wide range of concentrations, in some cases spanning over 8 orders of magnitude^105^ (Figure 8). AFFF is a common point source of PFAA contamination.^18^ This foam is frequently used during firefighter training and in response to fire-induced emergencies, and it contains a mixture of PFAAs.^106^ As a result, AFFF was found to contribute significantly to the deposition of PFAAs in the environment. The concentration of an individual PFAA in AFFF can be as high as 20 mM, which is approximately three to four times the cmc of some PFAAs (PFHpA, PFOA).^17,18,107^ However, at AFFF impacted sites, reported PFAA concentrations in soils and water exhibit erratic behavior, showing little consistency across the literature. For example, PFOA concentrations in AFFF-impacted soils vary widely from <1 to 120 mmol kg^–1^.^108^ While various factors could contribute to this variation, including the time elapsed since the deployment of AFFF at the site and the analytical methods used for characterization, one crucial aspect to consider is the influence of the surrounding environmental conditions. As stated in section 3.1, the ionized state of the PFCA headgroup is dependent on the surrounding chemistry of the media (i.e., pH, dissolved ions). Such variation in the ionization of the headgroup can result in drastically different interfacial adsorption behavior of the PFCA molecule, affecting its transport, adsorption and micellization.^48^ Nonetheless, the ongoing debate within the PFAS community revolves around the equilibrium acid dissociation constant (*K*_a_) of many PFAAs and how this factor will affect its environmental fate.^3^ Overlooking the chemistry of the sampling media could lead to misguided conclusions regarding experimental data; therefore, it is important to consider such fundamental parameters when conducting field sampling of PFAAs.

In contrast to soil, the concentrations of PFAAs in both surface water and groundwater at AFFF-impacted sites are comparatively low. Reported PFAA concentrations progressively diminish to trace levels during their transport through the environment via water pathways, ultimately leading to their deposition into the ocean (Figure 8). Often times, both surface and groundwater concentrations are reported to be less than 1 mM,^106,109^ before eventually reaching concentrations at approximately 2 × 10^–9^ mM in oceanic sinks^110^ (Figure 8). One explanation for this is by virtue of the various interactions and mechanisms described in this article that PFAAs demonstrate a great affinity for interfaces and high mobility throughout the environment. These characteristics of amphiphilic molecules raise questions about our conventional understanding of PFAS accumulation in the environment. Such large uncertainty in the estimation of the PFAA concentration in water bodies hinders our ability to fully comprehend the environmental impact of PFAS. Because of the low amounts in the environment combined with the high interfacial activity of the PFAAs, it is important to exercise caution when drawing conclusions about the environmental behavior of these molecules. Similar to the surrounding environmental conditions, it is important to consider how the chemical structure of the PFAAs will influence their dispersed state in the water column and correspondingly alter their interfacial adsorption behavior and transport properties.

According to fundamental chemistry principles, if the number of carbon atoms in the hydrophobic chain of a surfactant molecule is reduced, then the molecule itself becomes more water-soluble. This fundamental concept can explain numerous reports where short chain PFAAs (≤8 carbon atoms) are the predominate form of PFAA detected in water.^111−114^ On the other hand, long chain PFAAs (≥8 carbon atoms) are typically found to exist in soil media at much higher concentrations, relative to their short chain counterparts.^108,115^ These observations further demonstrate the significance of considering the chemical structure of PFAAs in understanding their behavior and distribution in different environmental compartments. Additionally, the headgroup of the PFAA will alter the ionization state of molecules in environmental media. PFAAs with a sulfonic acid headgroup e.g. PFOS, are likely to exhibit much different behavior relative to those with a carboxylic acid headgroup, e.g., PFOA and warrant further investigation.

The amphiphilic properties of PFAAs present challenges not only from a research perspective but also when crafting regulatory policy. Over the past two decades, we have realized the overwhelming presence of PFAS in the environment.^116^ This discovery, coupled with their potential adverse effects to human health, has put pressure on policymakers to regulate the use of these substances, leading to the development of a new generation of fluorosurfactants.^117−119^ As industry rushes to develop next generation PFAAs for a multitude of applications, it is critical to consider the unique life cycles of PFAAs with respect to their chemical makeup and the environments in which they will inevitably become integrated. For instance, the current generation of PFAAs is largely constituted of molecules with shorter fluoroalkyl chains (C ≤ 8), likely due to the lower serum half-lives of short chained PFAAs.^120,121^ Additionally, the potential for the biological degradation of PFAS appears to be more promising for compounds with shorter fluoroalkyl chains.^54^ However, short chained PFAAs exhibit high environmental mobility, easily traversing through soil media and rapidly contaminating water resources.^122^ The decreased hydrophobicity and increased water solubility of short chained PFAAs makes their removal from wastewater streams exceedingly difficult relative to longer chained PFAAs.^123,124^ Hence, it is critical for policymakers to consider the lifecycle implications originating from the underlying chemistry of PFAAs as they proceed with regulatory actions. We believe that the investigations of the dependence of inherent fundamental properties like p*K*_a_ on the interfacial characteristics of PFAAs will play a pivotal role in both the development of future surfactants for consumer products and for formulating regulations to minimize the environmental impacts of PFAS.
